# Target Vessel Versus Complete Revascularization in Non-ST Elevation Myocardial Infarction Without Cardiogenic Shock

**DOI:** 10.7759/cureus.23139

**Published:** 2022-03-14

**Authors:** Neeraj Pandit, Parag Rahatekar, Lokendra Rekwal, Dheerendra Kuber, Ranjit K Nath, Puneet Aggarwal

**Affiliations:** 1 Cardiology, Atal Bihari Vajpayee Institute of Medical Sciences (ABVIMS) and Dr. Ram Manohar Lohia (RML) Hospital, New Delhi, IND; 2 Cardiology, Anika Heart Center, Nagpur, IND; 3 Cardiology, Super Speciality Hospital, Mahatma Gandhi Memorial (MGM) Medical College, Indore, IND

**Keywords:** multivessel disease, target vessel revascularization, complete revascularization, percutaneous coronary intervention, non-st segment elevation myocardial infarction (nstemi)

## Abstract

Introduction

The role of complete revascularization (CR) vs target vessel revascularization (TVR) in non-ST-elevation myocardial infarction (NSTEMI) in patients without cardiogenic shock is still not established. In this study, we compared outcomes at one and six months among patients with NSTEMI with multivessel disease (MVD) undergoing CR vs TVR.

Methods

It was a prospective, observational study carried out among 60 NSTEMI patients with MVD (30 undergoing TVR and 30 CR) from October 2018 to November 2019. They were assessed at one and six months for primary and secondary outcomes.

Results

The mean age of the patients was 56.13 ± 9.23 years and both the groups were well matched with respect to age, gender, risk factors, and comorbidities. In the majority of patients, the target vessel was left anterior descending (LAD) followed by right coronary artery (RCA) and left circumflex (LCX) in both groups. The primary outcomes of death from any cause, non-fatal myocardial infarction, and the need for revascularization of the ischemia-driven vessel showed no significant difference at one and six months follow-up between the CR and TVR groups. However, the secondary outcomes of heart failure hospitalizations and angina episodes were significantly more in the TVR group than CR group at one month (6 vs 1, P=0.044), (8 vs 2, P=0.038) and six months (8 vs 2, P=0.038), (9 vs 2, P=0.02), respectively.

Conclusion

CR was associated with no difference in death from all-cause or future revascularization but significantly lesser secondary outcomes of heart failure hospitalizations and angina episodes as compared to TVR in NSTEMI without cardiogenic shock.

## Introduction

Current epidemiological data indicate non-ST-elevation myocardial infarction (NSTEMI) is a more frequent occurrence of myocardial infarction than ST-segment elevation myocardial infarction (STEMI) [1,2]. The evidence-based management of STEMI in eligible patients after primary percutaneous coronary intervention (PPCI) is complete revascularization (CR). However, the role of complete revascularization vs target vessel revascularization (TVR) only in NSTEMI is still not established. Among patients with NSTEMI who underwent coronary angiography, approximately one-third of patients have single-vessel disease while approximately one-half have a multivessel disease (MVD) [3]. Percutaneous coronary intervention (PCI) of the culprit vessel is usually the recommended management in both single and MVD [4]. However, the decision to perform an interventional procedure in the non-culprit artery in MVD in NSTEMI is still debatable. There is a lacuna in the available scientific literature on this subject. No large multicentre randomized clinical trial (RCT) has compared CR and TVR in NSTEMI without cardiogenic shock. CR is infrequent in clinical practice, and guidelines do not formally address this issue in detail [5].

Thus, the purpose of the present study is to compare outcomes at one month and six months in patients of NSTEMI with MVD undergoing complete revascularization vs target vessel revascularization only.

## Materials and methods

Study population

We conducted a prospective observational study among 60 NSTEMI patients with multivessel disease admitted at ABVIMS and Dr. RML Hospital, New Delhi, India from 2018 to 2019. We included consecutive adult patients (>18 years old) diagnosed as having NSTEMI with MVD on coronary angiography. Patients having >90% stenosis in two or more epicardial coronary arteries due to difficulty in identifying target vessel in these patients were excluded from the study. We also excluded patients who presented with cardiogenic shock.

Demographic data and history regarding onset, duration, course, progression of complaints, past history, risk factors, and medications history were obtained from the patient. They were clinically examined and routine blood tests, cardiac biomarkers were obtained. Electrocardiogram, an echocardiogram was done and patients diagnosed with NSTEMI were subjected to a coronary angiogram.

Angiogram and selection of target vessel

The coronary angiogram was reviewed and stenosis was assessed visually by a well-trained cardiologist. The target vessel was identified based on at least two of the following angiographic characteristics, i.e., intraluminal filling defect, plaque ulceration, plaque irregularity, dissection, or impaired flow [6] along with evidence of regional wall motion abnormality (RWMA) in the territory of the target vessel and electrocardiogram (ECG) leads showing significant ST-T changes.

Multivessel disease was defined as the presence of at least two hemodynamically significant narrowed (diameter stenosis of more than 70%) major epicardial arteries during angiography. In vessels with diameter stenosis of 50-70%, fractional flow rate (FFR) was done to determine hemodynamically significant lesions.

Revascularization strategies

Target vessel revascularization (TVR) was defined as revascularization of significant stenosis in the artery responsible for ischemia as identified by the above-mentioned criteria, whereas complete revascularization was defined as revascularization in all major significantly diseased epicardial vessels during the same hospitalization.

The decision regarding treatment strategy in the form of target vessel revascularization only or complete revascularization was decided by the operator on the basis of clinical and angiographic criteria [7] and patient preference and affordability.

In some patients, identifying the target vessel was difficult based on coronary angiographic and echocardiographic criteria (like global left ventricle hypokinesia). These patients were included in the CR group. Both the groups were given guideline-directed optimal medical therapy [8-10].

Study outcomes

Patients were assessed at one month and six months follow-up for outcomes. Primary outcomes included death from any cause, nonfatal myocardial infarction, and need for revascularization of ischemia-driven vessel whereas secondary outcomes included hospitalizations for heart failure (HF) and angina episodes. At each follow-up visit, data regarding the above-mentioned outcomes were assessed by the history, clinical examination, and ECG.

Statistical analysis

Data were analyzed by using the statistical package SPSS 13 software (SPSS Inc., Chicago, IL). The continuous and discrete variables were expressed as mean and standard deviation (SD). Differences were analyzed using Student’s t-test to compare two variables and continuous or discrete analysis of variance (ANOVA) when comparing more than two variables. The categorical variables were expressed as frequencies and percentages and compared using chi-square or Fisher’s exact test, depending on the frequency of expected events. Results were expressed as the two-tailed odds ratio (OR) with a 95% confidence interval (CI). Differences with a p-value of less than 0.05 were considered significant.

## Results

Our study included 60 patients with NSTEMI. Thirty patients underwent target vessel revascularization only and the remaining 30 underwent complete revascularization by PCI and drug-eluting stents. All the patients received guideline-directed optimal medical therapy.

The mean age of the patients was 56.13 ± 9.23 years and there were 43 males (71.7%) and 17 females (25.3%). There were no statistically significant differences between the TVR and CR groups in age, gender, risk factors, and comorbidities (Table 1). Also, the low-density lipoprotein cholesterol (LDL-C) levels were matched between the two groups. Serum creatinine levels in the CR group were significantly lower than in the TVR group (P=0.035).

**Table 1:** Baseline characteristics of study population TVR- target vessel revascularization, CR- complete revascularization, LAD- left anterior descending artery, LCX- left circumflex artery, RCA- right coronary artery, LDL-C - low-density lipoprotein cholesterol.

Patients	TVR group	CR group	P-value
Number	30	30	1
Age	57.53 ± 8.98	54.73 ± 9.40	0.24
Sex (MALE/FEMALE)	21/9	22/8	0.77
Hypertension	13	13	1
Diabetes	5	8	0.35
Current Smokers	11	11	1
Chronic obstructive airway disease	4	3	0.68
Dyslipidemia (LDL-C ≥ 100 mg/dl)	13	14	0.79
Hypothyroidism	1	0	0.31
Serum creatinine (mg/dL)	1.18 ± 0.30	1.02 ± 0.26	0.03
Left ventricle ejection fraction (%)	53.83 ± 9.25	48.67 ± 9.73	0.04
Echocardiogram hypokinesia			0.17
No hypokinesia	20	14	
LAD territory hypokinesia	5	7	
LCX territory hypokinesia	1	3	
RCA territory hypokinesia	2	6	
Global hypokinesia	2	0	

The mean left ventricular ejection fraction (LVEF) in the TVR group was 53.83 ± 9.255% whereas it was 48.67 ± 9.732% in the CR group with the majority of the patients having normal LVEF, i.e., 34 patients (20 in TVR group and 14 in CR group). Left anterior descending artery (LAD) territory hypokinesia was observed in a total of 12 (20%) patients (five in TVR group and seven in CR group). LAD territory hypokinesia was more commonly seen than left circumflex artery (LCX) or right coronary artery (RCA) territory hypokinesia.

The majority of the patients had LAD as their target vessel, i.e., 13 (43.3%) in the TVR group and 14 (46.7%) in the CR group followed by RCA [9 (30%) each in TVR and CR groups] and then LCX [7 (23.3%) in TVR and 4 (13.3%) in CR group]. Left main (LM) was the target vessel in two (6.7%) patients in TVR and one (3.3%) in the CR group (Table 2).

**Table 2:** Distribution of target vessel in target vessel and complete revascularization group TVR- target vessel revascularization, CR- complete revascularization.

Target vessel	TVR group (N=30)	CR group (N=30)	P-value
Left Anterior Descending artery	13 (43.3%)	14 (46.7%)	0.795
Left Circumflex Artery	7 (23.3%)	4 (13.3%)	0.317
Right Coronary Artery	9 (30%)	9 (30%)	1.0
Left Main Artery	2 (6.7%)	1 (3.3%)	0.554
Other	0	2 (6.7%)	0.150

In our study, the primary outcomes of death from any cause, nonfatal myocardial infarction, and the need for revascularization of the ischemia-driven vessel showed no statistically significant difference at one and six months between the CR and TVR groups (Table 3).

**Table 3:** Primary outcome in study population at one-month and six-month follow-up TVR- target vessel revascularization, CR- complete revascularization, MI- myocardial infarction.

Outcome	1-month follow-up		P-value	6-month follow-up		P-value
	TVR group	CR group		TVR group	CR group	
Death from Any Cause	0	0	1.0	1	0	0.31
Non-Fatal MI	0	0	1	1	1	1
Need for Revascularization of Ischemia-Driven Vessel	1	0	0.313	0	1	0.313

The secondary outcomes of heart failure hospitalizations were significantly more in the TVR group than CR group at one month [6 (20%) patients vs 1 (3.3%) patients, P=0.044] and six months [8 (26.7%) patients vs 2 (6.7%) patients, P=0.038]. Also, the secondary outcomes of angina episodes were significantly more in the TVR group than CR group at one month [8 (26.7%) patients vs 2 (6.7%) patients, P=0.038] and six months [9 (30%) patients vs 2 (6.7%) patients, P=0.02] follow-up period (Table 4).

**Table 4:** Secondary outcome in study population at one-month and six-month follow-up TVR- target vessel revascularization, CR- complete revascularization, HF- heart failure.

Outcome	1-month follow-up		P-value	6-month follow-up		P-value
	TVR Group	CR Group		TVR Group	CR Group	
HF Hospitalization	6	1	0.04	8	2	0.04
Angina Episode	8	2	0.04	9	2	0.02

## Discussion

Revascularization of non-infarct-related artery probably improved myocardial ischemia causing better clinical outcomes in the multivessel PCI group in some studies, however, the literature is still debatable in patients with no cardiogenic shock in NSTEMI.

Shishehbor et al. [11] analyzed 1240 patients with acute coronary syndrome (ACS) with multivessel disease, 479 of whom underwent multivessel and 761 received CR PCI with a bare-metal stent and found no differences in periprocedural myocardial infarction or acute kidney injury between the groups. In a median follow-up of 2.3 years, CR PCI was associated with a better prognosis (HR: 0.80; 95% CI: 0.64-0.99; p=0.04), related to reduction in the need for repeat revascularization. Our study differs from this study as all patients underwent PCI with drug-eluting stents and the need for revascularization of the ischemia-driven vessel showed no statistically significant difference between the two groups after one and six months. But secondary outcomes of HF hospitalizations and angina episodes were significantly higher in the TVR group than in the CR group.

Another study by Lee et al. [12] analyzed 366 patients; 187 were assigned to the target vessel PCI group and 179 to the multivessel PCI group. TVR was associated with higher major adverse cardiac events (MACE) (32.6% vs 19.6%; p=0.003), due to repeat revascularization (28.9% vs 13.4%; p < 0.001). Death from all causes and myocardial infarction rates were similar between the two groups. Multivariate analysis found CR PCI as an independent predictor of favorable prognosis in 36±6.5 month follow-up (HR: 0.50; 95% CI: 0.30-0.85; p=0.01). The majority of outcomes of our study correlate with this study except for the primary outcome of the need for revascularization of the ischemia-driven vessel which showed no statistically significant difference. However, our study sample size was small and the follow-up period was till six months only. Thus a longer duration of follow-up and sample size may be required to draw any significant conclusion on primary outcomes.

A study by Bauer et al. [13] in Euro Heart Survey registry studied in-hospital outcomes of 1920 patients with NSTEMI and MVD. TVR was performed in 1186 and CR in 734 patients and found CR to be associated with a more periprocedural myocardial infarction (1.8 vs 5.3; p<0.0001). Our study however did not show any significant difference of events of non-fatal or fatal MI between the two groups.

Another study by Kim et al. [14] analyzed 1919 NSTEMI patients, 908 of whom underwent TVR and 1011 multi-vessel CR PCI. The study allowed staged revascularization in index hospitalization and found a significantly higher (2.9% vs 1.4%; p=0.025) mortality in the TVR group with similar periprocedural complications, cardiogenic shock and acute kidney injury. After a one year follow-up, MACE were found higher (18.6% vs 12.9%; p=0.002) in TVR group due to higher mortality (6.4% vs 3.5%; p=0.009) and repeat myocardial infarction (2.1% vs 0.6%; p=0.012) compared to the CR PCI group. However, repeat revascularization of culprit (1.7 vs 0.6; p=0.052) and non-culprit vessels (4.6% vs 2.8%; p=0.075) were non-significant but higher in CR group. Our study was similar in allowing staged procedures in our patients but there was no significant difference among the primary outcomes between the groups. Secondary outcomes were significantly more in the TVR group compared to the CR group.

A significant more secondary outcome of HF hospitalizations and angina episodes in the TVR group compared to the CR group in our study could be explained by some factors like the untreated diseased vessels in the target vessel-only PCI group might progress and may be responsible for more angina events and disease progression in non-culprit vessels in TVR group could lead to myocardial dysfunction and HF events. Also, revascularization of a non-infarct-related artery in the CR group would probably improve myocardial ischemia and left ventricular EF and thus cause less frequent heart failure events.

The majority [11,12,14] of the observational studies suggested complete revascularization while others showed no additional benefit as compared to target vessel revascularization [13]. No RCT has compared the complete vs target vessel revascularization in patients with NSTEMI. A meta-analysis found decreased MACE with complete revascularization in patients with NSTEMI and multivessel disease [15]. The current guidelines [16], also have not been conclusive in determining the ideal treatment strategy among these patients and suggested an individualized approach.

Limitations of the study

It was an observational study with a small sample size. The culprit vessel was selected on the basis of angiographic images and did not involve the use of intracoronary imaging like intravascular ultrasound (IVUS) or optical coherence tomography (OCT). Moreover, in patients undergoing complete revascularization, the study did not differentiate patients undergoing “one-sitting PCI” vs. “staged PCI” because the majority of patients in the CR group in our study underwent “one sitting PCI”.

## Conclusions

In this study, patients having non-ST elevation myocardial infarction with multivessel disease undergoing complete revascularization showed no significant difference when compared to target vessel revascularization only with respect to primary outcomes of death from any cause, non-fatal myocardial infarction, and need for revascularization of ischemia-driven vessel. However, patients undergoing complete revascularization had better outcomes when compared to target vessel revascularization for secondary outcomes of heart failure hospitalizations and angina episodes. Thus complete revascularization of significant lesions may be reasonable.
